# Monitoring respiration and oxygen saturation in patients during the first night after elective bariatric surgery: A cohort study

**DOI:** 10.12688/f1000research.11519.2

**Published:** 2017-06-23

**Authors:** Liselott Wickerts, Sune Forsberg, Frederic Bouvier, Jan Jakobsson

**Affiliations:** 1 Department of Anaesthesia, Norrtälje hospital, TioHundra AB, Norrtälje, Sweden; 2 Department of Clinical Sciences, Karolinska Institutet, Danderyd Hospital, Stockholm, Sweden; 3 Department of Anaesthesiology and Intensive Care, Norrtälje Hospital, Norrtälje, Sweden; 4 Department of Clinical Science and Education, Södersjukhuset, Karolinska Institutet, Stockholm, Sweden; 5 Norrtälje hospital, TioHundra AB, Norrtälje, Sweden; 6 Department of Anaesthesia and Intensive Care, Institution for Clinical Sciences, Karolinska Institutet, Danderyd Hospital, Stockholm, Sweden

**Keywords:** obesity, bariatric surgery, general anaesthesia, postoperative polygraphy

## Abstract

**Background**: Obstructive sleep apnoea and obese hypoventilation is not uncommon in patients with obesity. Residuals effect from surgery/anaesthesia and opioid analgesics may worsen respiration during the first nights after bariatric surgery. The aim of this observational study was to monitor respiration on the first postoperative night following elective bariatric surgery.

**Methods**: This observational study aimed to determine the incidence and severity of hypo/apnoea in low risk obsess patients undergoing elective bariatric surgery in general anesthaesia. Patients with known or suspected sleep respiratory disturbances was not included. ESS was scored prior to surgery. Oxygen desaturation was analyzed by continuous respiratory monitoring. Mean oxygen saturation (SpO2), nadir SPo2, apnoea/hypopnea index and oxygen desaturation index was assess by standard tools.

**Results**: 45 patients were monitored with portable polygraphy equipment (Embletta, ResMed) during the first postoperative night at the general ward following elective laparoscopic bariatric surgery. The prop ESS was 0-5 in 22, 6-10 in 14 and 11-16 in 6 of the patients studied (missing data 3). Mean SpO2 was 93%; 10 patients had a mean SpO2 of less than 92% and 4 of less than 90%. The lowest mean SpO2 was 87%. There were 16 patients with a nadir SpO2 of less than 85%, lowest nadir SpO2 being 63%. An Apnoea Hypo/apnoea Index (AHI) > 5 was found in 2 patients only (AHI 10 and 6), and an Oxygen Desaturation index (ODI) > 5 was found in 3 patients (24, 10 and 6, respectively). 3 patients had more prolonged (> 30 seconds) apnoea with nadir SpO2 81%, 83% and 86%. ESS score and type of surgery did not impact on respiration/oxygenation during the observation period.

**Conclusions**: A low mean SpO2 and episodes of desaturation were not uncommon during the first postoperative night following elective bariatric surgery in patients without history of night time breathing disturbance. AHI and/or ODI of more than 5 were only rarely seen. Night-time respiration monitoring provided seemingly sparse additional information. Further studies are need to assess risk factors and potential impact of the desaturation episodes that occurs during sleep.

## Introduction

Obesity is on the increase in the western world and is associated with the development of several diseases. It is a major risk factor for cardiovascular disease and diabetes, two of the leading causes of death globally. Many efforts have been made in trying to treat the condition ( http://www.who.int/mediacentre/factsheets/fs311/en/). The adverse effects on pulmonary function are also well documented ^1^. With an increasing BMI, the risk for chronic daytime hypoventilation escalates, characterized by an arterial carbon dioxide pressure (PCO _2_) exceeding 45 mmHg ^1^. Complications include atelectasis, hypoxemia, pulmonary embolism and subsequent acute ventilation failure, and may evolve during perioperative and postoperative phases ^1^. Adverse effects are not concentrated to daytime; obesity is the most frequent predisposing factor of obstructive sleep apnoea syndrome (OSAS) ^1^. Changes in the breathing pattern and pulmonary function may indeed cause compromise of oxygenation and increased risk for oxygen desaturation.

The early postoperative period with residual anaesthetic and analgesic effects may put an obese patient that has just undergone a laparoscopic bariatric procedure at risk for respiratory compromise. It has been debated whether early care should be carried out in a high dependency ward or could be safely done in a general ward.

The present study aimed to monitor first postoperative night respiration, breathing patterns and oxygenation with sleep breathing equipment, in patients having undergone elective laparoscopic bariatric surgery. We wanted to assess whether we could define risk factors associated with hypo/apnoea and desaturation episodes.

## Methods

This is an explorative cohort study; the study protocol was approved by the ethical committee at Karolinska Institutet [Dnr 2015/118 – 31/1 La]. Patients were included in the study after having provided verbal and written informed consent. Patients with known sleep related respiratory disturbances, diagnosed or suspected obstructive sleep apnoea was excluded.

Each patient filled out a questionnaire to determine if there was a suspicion of OSAS preoperatively; the questionnaire included the Epworth Sleepiness Scale (ESS). Patients were monitored after surgery from 10 pm during the first postoperative night, until 6 am the next morning, with a portable OSAS breathing pattern monitor Embletta (ResMed). The registration included information about airflow from a nasal cannula, thoracic respiratory movements by an elastic band around the thorax and percutaneous O2 saturation and heart rate from a pulse oximeter.

Apnoea was classified in accordance to the American Academy of Sleep Medicine (AASM) as a drop in the peak signal excursion by ≥ 90% of pre-event baseline air flow signal. The duration of the ≥90% drop in sensor signal must be ≥10 seconds. Hypopnea was classified by as a drop in the peak signal excursion by ≥ 30% of pre-event baseline. The duration of the ≥ 30% drop in signal excursions must be ≥ 10 seconds (Berry *et al.*, 2012) ^2^.

All patients had anaesthesia and postoperative pain management in accordance to the routines of the department. All patients received premedication with 2 tablets of slow release 655mg paracetamol and 10 mg slow release oral oxycodone prior to surgery. Patients were preoxygenated by FiO2 1.0 and by CPAP of 6 cm H20 in the anaesthetic machine Aisys (GE Healthcare). Anaesthesia was induced with remifentanil target control infusion (TCI), set at a target of 6.0 ng/ml. After 90 seconds the patient was put to sleep with a bolus injection of propofol 2–3 mg/kg. When the patient got apnoeic the ventilation mode was changed to pressure control ventilation-volume guaranteed (PCV-VG), and the patient received neuromuscular blocker rocuronium, followed by endotracheal intubation. Anaesthesia was maintained with sevoflurane and remifentanil titrated to clinical signs of adequate anaesthesia, and with a BIS (Medtronic, Covidien BIS LoC 2 Channel) value between 25 and 50. All patients received postoperative nausea and vomiting (PONV) prophylaxis with betamethasone, ondansetron and droperidol. 10 – 15 mg of morphine was administered at the beginning of surgery, as a start dose for the postoperative pain relief regime. The patient had laparoscopic surgery, gastric bypass or sleeve gastrectomy, as decided by the surgeon. Postoperative care was provided in accordance to routines; fentanyl 25–50 micrograms was used as rescue analgesia and a further 1–5 mg morphine administered as needed. Postoperative respiratory care included oxygen supplementation to satisfactory saturation and once per hour blowing in a T-piece with a one-way valve mouthpiece (Intersurgical). Patients were transferred to the general ward when fully awake and with stable vital signs for 30 minutes. No intervention was done apart from the night breathing monitoring.

### Statistics

All data is presented as mean and standard deviation. The breathing data was evaluated in accordance to standard assessment,

- the AHI was calculated as hypo and/or apnoea/hour
- the ODI was calculated as oxygen saturation decrease of > 4 for 30 seconds/hour

## Results

There were 52 patients initially included in the study, but 6 were excluded as they had a diagnosis of OSAS and 1 further patient was excluded as the procedure became merely a diagnostic laparoscopy. Forty-five patients were included in the study.

The mean age for the 45 patients studied was 39 (ranging from 19 – 68 years), and the mean BMI was 37 (ranging from 32 – 53). The preoperative ESS scale screening showed that 22 of our patients had an ESS 0-5, 14 had a score of 6 – 10 and 6 patients had ESS of more than 10. Highest ESS was 16 in one 26 year old female patient with a BMI of 41.

Surgery and anaesthesia was uneventful, mean duration of surgery was 54 minutes (ranging from 27 – 97 minutes) and mean duration of anaesthesia was 108 (ranging from 65 – 217 minutes). Most patients (n=35), had a sleeve gastrectomy and 10 had a gastric bypass procedure.

Mean time between end of anaesthesia and start of the polygraphy was 611 ± 122 minutes and mean duration of polygraphy monitoring was 463 ± 51 minutes. 

Mean saturation (SpO2) during the polygraphy was 93% (ranging from 87 – 97). There were 10 patients with a mean SpO2 of less than 92% and 4 with mean of less than 90, with the lowest mean SpO2 being 87%. There were 16 patients with a nadir SpO2 of less than 85%, lowest nadir SpO2 being 63%.

Only 2 patients had an AHI > 5; (AHI 10 and 6). Both underwent sleeve gastrectomy. They also had an ODI > 5 (10 and 24, respectively). These patients had mean saturation 88% and 91% during the registration and SpO2 nadir of 79% and 81%.

In total, 3 patients had an ODI > 5 (24, 10 and 6, respectively). Additionally, 3 patients had more prolonged (> 30 second) apnoea with nadir SpO2 81%, 83% and 86%.

Changes in respiration and oxygenation did not differ between the 3 ranges of ESS scores, see Table 1.

**Table 1.** Demographics for the patients studied; mean ± SD. GBP: gastric bypass; Sleeve: sleeve gastrectomy; f: female; m: male.

	GBP n=10	Sleeve n=35
Age ( *years*)	37 ± 15	49 ± 13
Gender ( *f/m*)	9/1	30/5
BMI	36 ± 3	37 ± 5

We could further not see any difference apart from a shorter duration of anaesthesia between the surgical procedures (See Table 2 and Table 3)

**Table 2.** Main findings; mean ± SD. GBP: gastric bypass; Sleeve: sleeve gastrectomy.

	GBP n=10	Sleeve n=35
Duration of anaesthesia *min.*	125 ± 20	103 ± 29 *
Duration of Surgery *min.*	63 ± 16	51 ± 18
Duration in the PACU *min.*	308 ± 89	355 ± 197
Time until start of registration *min.*	615 ± 129	609 ± 121
Duration of registration *min.*	461 ± 41	463 ± 55
Mean SpO2	93.1 ± 3.0	92.8 ± 2.1
Nadir SpO2	85.3 ± 9	84.3 ± 6
Max duration nadir SpO2	3 ± 7	9 ± 25
AHi	0.1 ± 0.3	0.7 ± 2
ODi	0.1 ± 0.3	1.5 ± 4.5

**Table 3.** Respiration and oxygenation in relation to the preoperative ESS score.

	ESS 0-5 (n=22)	ESS 6-10 (n=14)	ESS > 10 (n=6)
Age (years)	36 ± 13	40 ± 10	43 ± 18
BMI	35 ± 5	36 ± 4	38 ± 3
Mean SpO2	92 ± 2.4	93 ± 2.1	93 ± 1.9
Nadir SpO2	85 ± 6.1	84 ± 9.5	84 ± 5.1
AHi	0.7 ± 2.3	0.1 ± 0.4	0.2 ± 0.4
ODi	1.7 ± 5.3	0.4 ± 1.1	0

The one patient with an ESS of 16 did not show and hypo or apnoeas during the observation period, and had a mean SpO2 of 92 and nadir Spo2 of 79%.

Raw sleep monitoring data from 45 patients that was used as a basis for the findings in this study Click here for additional data file. Copyright: © 2017 Wickerts L et al. 2017 Data associated with the article are available under the terms of the Creative Commons Zero "No rights reserved" data waiver (CC0 1.0 Public domain dedication).

## Discussion

We found, somewhat surprisingly, only very minor respiratory disturbances in the cohort of patients having undergone elective bariatric surgery. No patients had a hypopnea index above thirty - only 2 had an AHI above 5. A majority of patients had low oxygen saturation of around 93%, and short episodes of saturation below 85% were not uncommon. Thus, the main finding is mild hypoxia and episodes of desaturation, but hypo/apnoea monitoring does not provide much additional information. Signs of more pronounced airway compromise causing hypo/apnoea were only rarely seen. Low mean saturation and desaturation episodes were however not uncommon. The preoperative ESS score did not help predict differences in oxygenation. All our patients had no further complications.

The risk for postoperative hypoxia has been known for long. Jones *et al.* published a review in 1990 addressing the risk for low oxygenation during the early postoperative period, and its multifactorial etiology ^3^. Low saturation and mild hypoxia has also been reported in previous studies and the risk in bariatric surgery must be acknowledged ^4^.

We are not aware of any previous study explicitly monitoring hypo/apnoea during the first postoperative night after bariatric surgery Zaremba *et al.* studied polysomnography in patients during the early postoperative course, while patients were still in the PACU ^5^. They found that 64% of the 33 patients with complete postoperative polysomnography data had signs of sleep-disordered breathing with an AHI greater than 5/h early after recovery from anaesthesia. The respiratory response to hypoxia and hypercapnia caused by airway obstruction is compromised following major surgery ^6^. Chung *et al.* studied respiration during the preoperative stage and the first, second and third postoperative nights ^7^. Our results are in line with these results, first night after surgery. Female patients and patients with no or mildly compromised nocturnal breathing showed only minor changes during the first postoperative night in Chung *et al’s* study as well as in ours. Age, preoperative respiration disturbance and smoking were found to be risk factors for hypo/apnoea ^8^. 

We used a standard portable breath and saturation monitor, the Embletta system. The portable systems have been shown to be accurate tool for assessment of sleep apnoea ^9^. We did not include EEG monitoring and we did not attach the abdominal movement tracing. Merely the nasal flow, thoracic and saturation was recorded. AHI scores were > 5 for mild, > 15 for intermediate and > 30 for significant airway compromise, or “sleep apnoea”. We assessed SpO2 < 94 as mild hypoxia. The monitoring was initiated during the first evening, an average of ten hours after the end of anaesthesia. It should also be acknowledged that the mean BMI in our cohort was 37, versus s mean BMI of 44 in the US studies ^5^.

Nocturnal oxygen desaturations are not uncommon during the first postoperative night, also in patients undergoing other surgical procedures. Shirmakana *et al.* found that oxygen desaturation was frequent in patients having undergone breast surgery ^10^. Bowdle looked at a mixed group of ambulatory surgery patients and also found an increase in hypo/apnoea and desaturation in the first night after surgery ^11^.

There are recent guidelines from the US association for sleep apnoea ^12^. Germany has also addressed the importance of adequate perioperative care of obese patients at risk for sleep apnoea ^13^. The preoperative evaluation by registration of hypo/apnoea is strongly recommended, however advice given around the early postoperative period is sparse. The ESS should of course also be used for screening ^14^. The waste majority of our patient had a “normal” ESS, and group of patients with ab ESS above 10 was small and did not provide any firm additional information.

To put our findings into perspective, our patients had a mean BMI of merely 37. Additionally, we had a majority (39 out of 45) of female patients, and BMI-associated AHI abnormalities are more commonly seen in males ^15^. The physical status, muscle strength and breathing capacity was not assessed or screened preoperatively. We did not use the abdominal movement monitoring band to avoid additional abdominal pain. All patients had multi-modal analgesia and opioids were administered as restrictively as possible to maintain adequate pain control. There is an obvious need for further studies looking for risk factors and also better determine potential risks associated to the desaturation episodes that are not uncommonly noticed.

In conclusion, we have found that elective “low risk” obese patients that have had uncomplicated laparoscopic bariatric surgery, have low saturation during the first postoperative night and may experience episodes of oxygen saturation of less than 85%, but hypo/apnoea is rare and monitoring of obstruction seems not to be of major value. The potential clinical impact of the mild hypoxia and short episodes of desaturations requires further study.

We have followed the STROBE guidelines.

## Data availability

The data referenced by this article are under copyright with the following copyright statement: Copyright: © 2017 Wickerts L et al.

Data associated with the article are available under the terms of the Creative Commons Zero "No rights reserved" data waiver (CC0 1.0 Public domain dedication). 

**Dataset 1. Raw sleep monitoring data from 45 patients that was used as a basis for the findings in this study.** DOI, 10.5256/f1000research.11519.d161775 ^16^
