# Supplementary material for: Monitoring respiration and oxygen saturation in patients during the first night after elective bariatric surgery: A cohort study
Source: F1000Res. 2017 Jun 23;6:735. Originally published 2017 May 22. [Version 2] doi: 10.12688/f1000research.11519.2 (PMC5538033; doi:10.12688/f1000research.11519.2)
Supplement: Peer review history

# Peer review: Referee response for version 2

**DOI:** 10.5256/f1000research.12973.r23753

**Published:** 2017-07-31

**Authors:** Frances Chung

**Affiliations:** 1 University Health Network, Toronto Western Hospital, Department of Anesthesia, University of Toronto, Toronto, ON, Canada

We are happy with the revisions.

I have read this submission. I believe that I have an appropriate level of expertise to confirm that it is of an acceptable scientific standard.

# Peer review: Referee response for version 2

**DOI:** 10.5256/f1000research.12973.r24052

**Published:** 2017-07-06

**Authors:** Peter N. Benotti

**Affiliations:** 1 Geisinger Obesity Institute, Geisinger Medical Center, Danville, PA, USA

I have reviewed the revised submission and feel the authors have addressed all of my comments in a satisfactory manner.

I have read this submission. I believe that I have an appropriate level of expertise to confirm that it is of an acceptable scientific standard.

# Peer review: Referee response for version 1

**DOI:** 10.5256/f1000research.12443.r23655

**Published:** 2017-06-20

**Authors:** Frances Chung, Jean Wong

**Affiliations:** 1 University Health Network, Toronto Western Hospital, Department of Anesthesia, University of Toronto, Toronto, ON, Canada; 2 University Health Network, Toronto Western Hospital, Toronto, ON, Canada

This observational study sought to determine the incidence and severity of hypopnea or apnea on the first night after surgery, in patients who did not have a diagnosis of OSA undergoing elective laparoscopic bariatric surgery. They monitored 45 patients with a portable polygraphy during the first postoperative night. They report that oxygen desaturation and short episodes of desaturation occurred in some patients, but there were few patients with an AHI or ODI greater than five. They conclude that respiratory monitoring for low risk patients on the general ward provides little additional information.

 Although this is an interesting study, this manuscript can be improved by providing more details in the Methods and Results. In the Introduction, the authors state that they wished to assess risk factors associated with hypopnea or apnea and desaturation. However, the presence of co-morbidities is not mentioned and there is no analysis of the risk factors that were associated with the patients that experienced desaturation/apnea/hypopnea. The questionnaire that was used to determine the risk of OSAS is indicated to be Epworth Sleepiness scale. The validation of this scale to screen for sleep apnea is relatively modest. It is not clear whether patients with obstructive sleep apnea are excluded from the study after use of questionnaire. Is sleep apnea one of the exclusion criteria of the study? The results of the questionnaire to determine risk of OSAS should be reported, it is unclear whether the patients who participated in the study were at low risk or may be at risk or have undiagnosed OSAS.

 The definition of apnea/hypopnea should be stated. The explanation for the sample size should be added. The number of patients receiving oxygen on the ward should be added, especially as it is mentioned in the methods section that patients received oxygen supplementation postoperatively. In Tables 1 and 2, the authors compare gastric bypass vs. sleeve gastrectomy, however, the authors have not stated whether they expected a difference between these 2 groups. There are some grammatical errors in the manuscript.

We have read this submission. We believe that we have an appropriate level of expertise to confirm that it is of an acceptable scientific standard, however we have significant reservations, as outlined above.

# Response

**Published:** 2017-06-20

**Authors:** Jan Jakobsson

**Affiliations:** Department of Physiology and Pharmacology, Karolinska Institutet, Sweden

Dear referee,

Thank you for review and most appreciated/important comments:

Patients with known sleep related respiratory disturbances, diagnosed or suspected obstructive sleep apnea was excluded.

We used the ESS scale for screening, 22 of our patients had an ESS 0-5, 14 had a score of 6 – 10 and 6 patients had ESS of more than 10. Highest ESS was 16 in one 26 year old female patient with a BMI of 41. She had a mean SpO2 of 92% and a nadir SpO2 of 79. She did not show and hypo or apneas during the observation period. There are 3 patients where ESS is missing.

Apnea was classified in accordance to the American Academy of Sleep Medicine (AASM) as a drop in the peak signal excursion by ≥ 90% of pre-event baseline air flow signal. The duration of the ≥90% drop in sensor signal must be ≥10 seconds. Hypopnea was classified by as a drop in the peak signal excursion by ≥ 30% of pre-event baseline. The duration of the ≥ 30% drop in signal excursions must be ≥ 10 seconds (Berry *et al* 2012).

Berry RB, Budhiraja R, Gottlieb DJ, Gozal D, Iber C, Kapur VK, *et al.* Rules for scoring respiratory events in sleep: update of the 2007 AASM Manual for the Scoring of Sleep and Associated Events. Deliberations of the Sleep Apnea Definitions Task Force of the American Academy of Sleep Medicine. J Clin Sleep Med. American Academy of Sleep Medicine; 2012 Oct 15;8(5):597–619.

We have analysed the results also taking the ESS into account, but we failed to see any clear association between ESS and respiration/saturation see Table 3

Table 3

ESS 0-5

(n=22)

ESS 6-10

(n=14)

ESS > 10

(n=6)

Age (years

36 ± 13

40 ± 10

43 ± 18

BMI

35 ± 5

36 ± 4

38 ± 3

Mean SpO2

92 ± 2.4

93 ± 2.1

93 ± 1.9

Nadir SpO2

85 ± 6.1

84 ± 9.5

84 ± 5.1

AHi

0.7 ± 2.3

0.1 ± 0.4

0.2 ± 0.4

ODi

1.7 ± 5.3

0.4 ± 1.1

0

We did not know whether surgical procedure had an impact and therefore tested also for surgical technique.

We hope that these responses is in line with your expectations, an update manuscript is on the way.

Best regards

Jan Jakobsson, on behalf of the authors

# Peer review: Referee response for version 1

**DOI:** 10.5256/f1000research.12443.r23609

**Published:** 2017-06-20

**Authors:** Peter N. Benotti

**Affiliations:** 1 Geisinger Obesity Institute, Geisinger Medical Center, Danville, PA, USA

Hypoxemia in the immediate postoperative period is a well-known and feared complication after bariatric surgery. Risk factors for hypoxemia include patient age (likely because of the increase in closing volume with aging), loss of lung volume during induction and maintenance of general anesthesia, alterations in respiratory drive related to general anaesthetics and use of opiates, as well as the the presence of obstructive sleep apnea. ^1,2^  The exact incidence of hypoxemia and hypercarbia after bariatric surgery has not been well studied, but several studies in relatively small numbers of patients have documented worrisome levels of hypoxemia. ^2,3^

 Wickerts and co-workers ^4^ have studied 45 good risk bariatric surgery patients. The mean age of the cohort was 39, the mean BMI was 37, and patients with known Obstructive Sleep Apnea were excluded. They all underwent uncomplicated and brief laparoscopic foregut procedures for obesity and were studied for 8 hours with pulse oximetry during the first postoperative night. Study findings included 22% with a mean SaO2 < 92%, 9% with mean SaO _2_ < 90% (lowest mean SaO _2_ was 87%), and 36% with nadir SaO _2 _< 85% (lowest nadir SaO _2_ was 63%).  In addition, sleep disordered breathing events were documented in several patients.  No clinical events related to hypoxia or sleep disordered breathing are recorded.

 These findings are important as because they do document a small, but significant risk of potentially dangerous postoperative hypoxia even in low risk patients. Recently published guidelines recommend continuous monitoring with pulse oximetry in the early postoperative period. ^4 ^Guidelines for perioperative management of low risk patients as studied here are lacking.

 Additional studies of the epidemiology of perioperative hypoxemia and hypercarbia complicating bariatric surgery are needed in order to begin to identify potentially modifiable patient-specific risk factors and interventions such is inspiratory muscle training ^3^ which might reduce the risks of this potentially dangerous problem. The authors are encouraged to continue this survey and attempt to relate hypoxemia to risk factors. In addition, continued studies to include EKG monitoring with pulse oximetry might add to the clinical significance of these findings. The authors comment that the observed episodes of desaturation were brief, but they do not share any data regarding the duration of desaturations. I would be concerned about the possible clinical impact of 30 seconds of apnea in an unmonitored setting on the initial post operative night following bariatric surgery

 In this era of cost constraints and limited patient access to bariatric surgery, cost reduction strategies designed to encourage same day bariatric surgery procedures are popular. Improved ability to recognize and address risk factors for postoperative hypoxemia should be an important component of the selection process for accelerated care.

 The phrase that “hypoxia not only stops the engine, but destroys the machinery” has been attributed to JBS Haldane. Case reports of respiratory arrest and sudden unexplained fatality after bariatric surgery suggest that he was correct.

I have read this submission. I believe that I have an appropriate level of expertise to confirm that it is of an acceptable scientific standard, however I have significant reservations, as outlined above.

## References

[1] LumachiFMarzanoBFantiGBassoSMMazzaFChiaraGB: Relationship between body mass index, age and hypoxemia in patients with extremely severe obesity undergoing bariatric surgery. *In Vivo*.24(5) :775-7 20952748

[2] GallagherSFHainesKLOsterlundLGMullenMDownsJB: Postoperative hypoxemia: common, undetected, and unsuspected after bariatric surgery. *J Surg Res*.2010;159(2) : 10.1016/j.jss.2009.09.003 622-6 10.1016/j.jss.2009.09.003 20006346

[3] LlorénsJRoviraLBallesterMMorenoJHernández-LaforetJSantonjaFJCassinelloNOrtegaJ: Preoperative inspiratory muscular training to prevent postoperative hypoxemia in morbidly obese patients undergoing laparoscopic bariatric surgery. A randomized clinical trial. *Obes Surg*.2015;25(6) : 10.1007/s11695-014-1487-4 1003-9 10.1007/s11695-014-1487-4 25376434

[4] RaaffC.A.LGorter-StamM.A.Wde VriesNSinhaA.CBonjerH.JChungFCoblijnU.KDahanAvan den HelderR.SHilgevoordA.A.JHillmanD.RMargarsonM.PMattarS.GMulierJ.PRaveslootM.J.LReiberB.M.Mvan RijswijkASingh P.MSteenhuisRTenhagenMVandervekenO.MVerbraeckenJWhiteD.Pvan der WielenNvan WagensveldB.A: Perioperative Management of Obstructive Sleep Apnea in Bariatric Surgery: a Consensus Guideline. *Surg Obes Rel Dis. (in press)*. http://dx.doi.org/10.1016/j.soard.2017.03.022 10.1016/j.soard.2017.03.022 28666588

# Response

**Published:** 2017-06-20

**Authors:** Jan Jakobsson

**Affiliations:** Department of Physiology and Pharmacology, Karolinska Institutet, Sweden

Dear Referee

Thank you for your important and constructive comments on our paper. Bariatric surgery do increase and enhanced recovery pathways are not uncommonly used. The aim of our study was to assess respiration/oxygenation in "low risk patients" undergoing elective laparoscopic "low risk bariatric surgery" and my expectation was indeed that we should observe more. Still agree even the observations found should be interpreted in a context; avoiding patient risk. 

We did not monitor duration of desaturation unfortunately but the hypopnea time was registered and was in two patients prolonged. The portable device used did not log duration of desaturation. We did not monitor ECG and/or sign of myocardial ischemia. There were however no complaints of chest pain or any other signs/symptoms alerting us to suspect myocardial or other ischemic events.

We were in the group of patients not able to identify explicit risk factors.

The literature is extensive and we are more than happy to add suggested papers.

We are planning to continue our efforts looking a´t the first nigh respiratory events and will in these studies add ECG.

I hope this response can be seen as acceptable and further suggestions for future studies are most welcome.

Best regards

Jan Jakobsson, on behalf of the authors
